# Green Nanoparticle Synthesis in the Application of Non-Bacterial Mastitis in Cattle

**DOI:** 10.3390/molecules30061369

**Published:** 2025-03-18

**Authors:** Michał Motrenko, Agata Lange, Aleksandra Kalińska, Marcin Gołębiewski, Małgorzata Kunowska-Slósarz, Barbara Nasiłowska, Joanna Czwartos, Wojciech Skrzeczanowski, Aleksandra Orzeszko-Rywka, Tomasz Jagielski, Anna Hotowy, Mateusz Wierzbicki, Sławomir Jaworski

**Affiliations:** 1 Department of Nanobiotechnology, Institute of Biology, Warsaw University of Life Sciences, 02-786 Warsaw, Poland; s202889@sggw.edu.pl (M.M.); anna_hotowy@sggw.edu.pl (A.H.); mateusz_wierzbicki@sggw.edu.pl (M.W.); slawomir_jaworski@sggw.edu.pl (S.J.); 2 Animal Breeding Department, Warsaw University of Life Sciences, 02-786 Warsaw, Poland; aleksandra_kalinska@sggw.edu.pl (A.K.); marcin_golebiewski@sggw.edu.pl (M.G.); malgorzata_kunowska-slosarz@sggw.edu.pl (M.K.-S.); 3 Institute of Optoelectronics, Military University of Technology, 00-908 Warsaw, Poland; barbara.nasilowska@wat.edu.pl (B.N.); joanna.czwartos@wat.edu.pl (J.C.); wojciech.skrzeczanowski@wat.edu.pl (W.S.); 4 Department of Plant Physiology, Institute of Biology, 02-776 Warsaw, Poland; aleksandra_orzeszko_rywka@sggw.edu.pl; 5 Department of Applied Microbiology, Institute of Microbiology, Faculty of Biology, University of Warsaw, 00-927 Warsaw, Poland; t.jagielski@uw.edu.pl

**Keywords:** yeasts, *Prototheca* spp., mastitis, green synthesis, silver nanoparticles

## Abstract

This study explores the potential of silver nanoparticles (AgNPs) synthesized through an eco-friendly method using coffee extract to combat non-bacterial mastitis in dairy cattle. Mastitis, often caused by pathogens such as yeasts and algae like *Prototheca* spp., poses a challenge due to the limited efficacy of traditional antibiotics. This research utilized strains isolated from mastitis milk and assessed the nanoparticles’ physicochemical properties, antimicrobial efficacy, and impact on biofilm formation and microorganism invasion. AgNPs demonstrated a spherical shape with a mean hydrodynamic diameter of ~87 nm and moderate colloidal stability. Antimicrobial tests revealed significant growth inhibition of yeast and *Prototheca* spp., with minimal inhibitory concentrations (MICs) as low as 10 mg/L for certain strains. Biofilm formation was notably disrupted, and microorganism invasion in bioprinted gels was significantly reduced, indicating the broad-spectrum potential of AgNPs. The study highlights the nanoparticles’ ability to damage cell membranes and inhibit metabolic activities, presenting a promising alternative for managing infections resistant to conventional treatments. These findings suggest that green-synthesized AgNPs could play a pivotal role in developing sustainable solutions for mastitis treatment, particularly for pathogens with limited treatment options.

## 1. Introduction

Mastitis, inflammation of the mammary gland, is one of the most common health problems in dairy farming, having a significant impact on production efficiency and milk quality. The most characteristic clinical sign of mastitis is a mild to severe swelling of the udder, which will also be excessively warm to the touch and red. Handling the udder will generally cause discomfort to the cow. In severe cases, the body temperature will rise, and the milk produced by the cow will be watery and may contain clots, pus, or blood. The most common mastitis pathogens are found in the udder tissues, spreading from cow to cow (contagious pathogens) or in the herd’s surroundings (environmental pathogens), such as bedding materials, manure, and soil [1]. Contagious pathogens can live on the udder and transfer from infected to healthy teats during milking. The most common pathogens in this context are *Streptococcus agalactiae*, *Staphylococcus aureus*, and *Mycoplasma bovis* [2]. Environmental pathogens enter the mammary gland most often through mechanical damage, e.g., due to improper milking and stress, which lowers the immunity of animals. These bacteria are often present in soil, water, and animal excrement. The main pathogens in this group include *Escherichia coli*, *Klebsiella* spp., *Staphylococcus uberis*, *Enterobacter* spp., and *Pseudomonas aeruginosa* [3]. Bacteria dominate among the factors causing this disease, but the role of non-bacterial pathogens, such as viruses, fungi, and protozoa, is also gaining more attention. Their presence can lead to inflammation and significantly complicate treatment processes. Yeast-induced mastitis most often occurs due to antibiotic therapy or veterinary procedures that disrupt the natural microbiota of the udder, creating favorable conditions for the growth of yeasts such as *Candida* spp., *Cryptococcus* spp., *Saccharomyces* spp., *Rhodotorula* spp., and *Trichosporum* spp. [4]. Yeast infection is more difficult to treat than bacterial mastitis because yeasts naturally resist antibiotics. These infections can be chronic, leading to a long-term deterioration of milk quality and irreversible damage to udder tissue. In such cases, it is crucial to use antifungal agents and implement preventive measures, including improving environmental hygiene and reducing unnecessary use of antibiotics.

*Prototheca* spp. are rare but increasingly common and underestimated causative agents of mastitis in dairy cows [5,6]. They are non-photosynthetic algae that naturally occur in the environment, mainly in soil and water. In the case of mastitis, *Prototheca* causes chronic, difficult-to-treat infections that lead to reduced milk yield and increased somatic cells, reducing milk quality. *Prototheca* is an opportunistic agent because the microorganism must adapt to heterotrophic conditions after losing its ability to photosynthesize. Its pathogenicity is revealed mainly in specific circumstances, especially in animals with weakened immune systems, where infection can develop despite the general resistance of the organism. These factors make *Prototheca* capable of causing infections, including mastitis in cattle, in appropriate environmental conditions [5]. A characteristic feature of *Prototheca* infections is their resistance to most currently available therapies [7]. The low efficacy of conventional treatments, combined with the extremely rare cases of spontaneous recovery, makes selection the preferred method of controlling and preventing the spread of infection in herds [8]. *Prototheca* spp. can survive standard chemical disinfection processes and a wide range of temperatures, including those used during pasteurization. This resistance allows them to persist in the environment for long periods of time, which may increase the risk of transmission to animals [9].

Treating mastitis caused by microorganisms such as *Prototheca* and yeast is more difficult than in cases of bacterial infections, mainly because these microorganisms do not respond to typical antibiotics. This necessitates the use of alternative antimicrobial agents. One promising approach involves using alternative antimicrobial agents with broad-spectrum activity against various pathogens. Recent studies have highlighted the potential of nanomaterials, particularly metallic nanoparticles, due to their unique physicochemical properties and antimicrobial mechanisms. Silver nanoparticles (AgNPs) are increasingly being investigated as a potential alternative to antibiotics, mainly due to their antibacterial [10], antiviral [11], and antifungal properties [12]. Nanoparticles are characterized by multi-level mechanisms of action, including perforation and damage of the membrane and cell wall, induction of oxidative stress, inhibition of protein synthesis, and DNA damage [13,14,15,16]. AgNPs demonstrate activity against various microorganisms, including Gram-positive and Gram-negative bacteria, viruses, fungi, and yeasts. The increasing demand for silver nanoparticles (AgNPs) every year motivates the search for alternative production methods. Silver nanoparticles produced by green synthesis methods are becoming more and more common. Green synthesis of silver nanoparticles (AgNPs) is an ecological process that uses natural, environmentally safe raw materials and methods instead of toxic chemicals. In this method, plants, microorganisms (bacteria, fungi), and various substances of biological origin, such as plant extracts, enzymes, or biopolymers, are used to reduce silver ions and stabilize the produced nanoparticles [17]. The phenolic compounds, such as caffeine and tannins present in the coffee extract, act as both reducing and stabilizing agents. One of the agents with which silver nanoparticles can be synthesized by green synthesis is *Coffea arabica* seed extract [18] or extract of spent coffee [19]. Moreover, the nanoparticles synthesized with the use of coffee are characterized by antimicrobial properties [20,21], albeit nanoparticles synthesized in this method have not yet been tested against *Prototheca* spp. and mastitis-associated yeast.

In this manuscript, we describe the effect of AgNPs obtained by automated synthesis using coffee extract, on the viability, invasion, and biofilm formation of yeast and algal pathogens responsible for mastitis development.

## 2. Results

### 2.1. Indetification of Microorganisms

The analysis focused on identifying previously isolated strains of yeasts using the MALDI-TOF MS system. This method allowed for precise identification based on mass spectrometry profiles, comparing the spectra obtained from the strains with existing databases. The identified yeast strains included *Candida albicans*, *Pichia fermentans*, *Pichia kudriavzevii*, *Wickerhamomyces anomalus*, and *Wickerhamiella pararugosa* (Table 1). The MALDI-TOF MS system is widely recognized for its reliability and speed, making it a preferred tool for yeast identification in clinical and research laboratories.

In contrast, the algal strains of *Prototheca bovis* could not be identified using the MALDI-TOF MS system as comprehensive reference spectra for this genus are not yet available in the database. Instead, *Prototheca* was identified through genetic analysis, specifically partial sequencing of the cytochrome b gene. In this study, three isolates were analyzed, *Prototheca* isolate 1 (PRO3), isolate 2 (PRO7), and isolate 3 (PRO32), with each distinguished by variations in the cytochrome b gene sequence (Table 2). Detailed results are presented in the Supplementary Material.

### 2.2. Physicochemical Analysis of Nanoparticles

**Figure 1:**
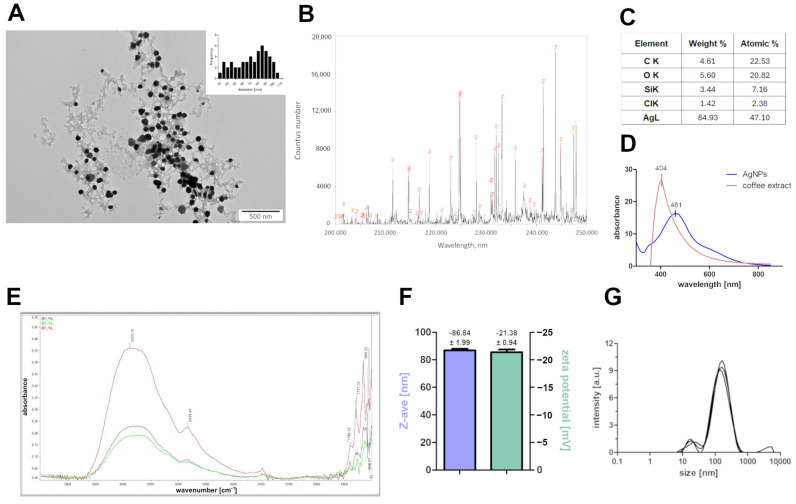
Physicochemical analysis of AgNPs. (**A**) TEM visualization with histogram of size distribution, (**B**) LIBS, (**C**) EDS analysis, (**D**) UV-Vis analysis, (**E**) FTIR-ATR, (**F**) zeta potential and Z-average graph, and (**G**) size distribution of AgNPs measured by DLS. Each analysis was carried out with a minimum of three replicates.

Figure 1 presents the results of physicochemical analysis. The obtained AgNPs showed a spherical shape. The hydrodynamic diameter of the obtained AgNPs was 86.84 ± 1.99 nm based on DLS analysis. The zeta potential of AgNPs was −21.38 ± 0.94 mV. Laser emission spectroscopy (LIBS) enabled us to determine the elemental composition of AgNPs. The results indicate a high Ag content, although C was also present (near the 227 and 247 nm peaks), which may be related to the presence of coffee extract. This also coincides with results from FTIR analysis, where peaks corresponding with groups like -OH (3353 cm^−1^), -CH/CH2 (2935 cm^−1^), C=O attributed to carboxylic acids (1760, 1717 cm^−1^), and at 1665 cm^−1^ C=O (ketones) or C=C (alkenes) were observed. The residues of the coffee extract were also confirmed by EDS analysis, in which, in addition to Ag, there were also C, O, Si, and Cl. The presence of silicon is caused by the background on which the nanoparticles were applied. UV-Vis analysis of the synthesized nanoparticles showed a broad peak between 400 and 500 nm.

### 2.3. Minimal Inhibitory Concentration (MIC)

The MIC values of silver nanoparticles (AgNPs) for various microorganism strains are presented in Table 3. The results indicate the minimum concentration of AgNPs that inhibited the growth of each strain. In general, the MIC values were the same for all microorganisms tested.

The table presents the MIC results for the individual microorganisms at different concentrations of AgNPs. The results showed no growth at higher concentrations of AgNPs (≥20 mg/L). In contrast, growth was observed at lower concentrations of silver nanoparticles, with most strains showing growth at concentrations starting from 10 mg/L.

### 2.4. Minimal F/A Concentration (MFC/MAC)

The MFC and MAC results show the minimum concentrations of silver nanoparticles (AgNPs) necessary to achieve inhibited growth effects on each microorganism. Table 4 displays the observed growth response at different AgNPs concentrations.

Table 4 presents the MFC results, demonstrating that higher concentrations of AgNPs effectively inhibited the growth of all tested yeasts. Similarly, the MAC values indicate the minimal concentration of AgNPs necessary to inhibit the growth of *P. bovis* isolates. At concentrations of 20 mg/L or higher, all tested microorganisms showed complete growth inhibition. However, at lower concentrations, variability in sensitivity was observed among the different strains. Strains such as *C. albicans*, *P. bovis* Isolate 3, and *W. anomalus* displayed growth persistence at 20 mg/L, indicating higher resistance. The remaining microorganism strains exhibited growth up to 10 mg/L.

### 2.5. Viability Analysis

**Figure 2:**
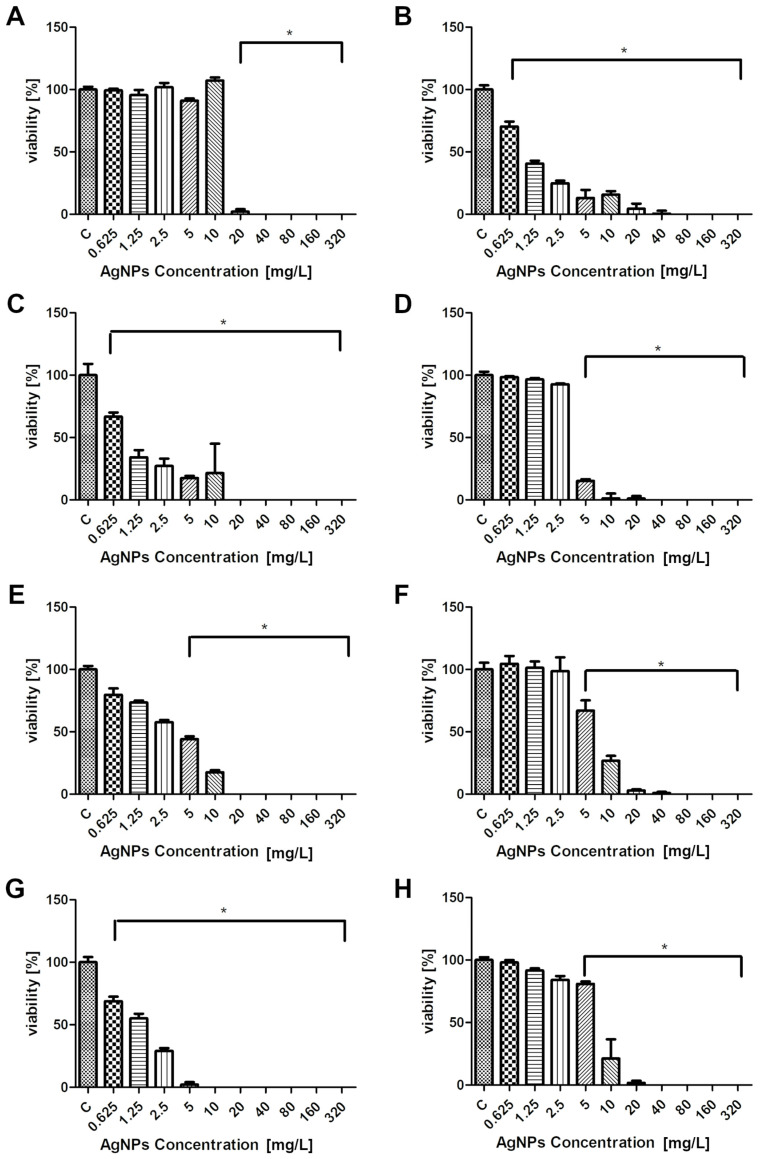
Viability analysis of tested microorganisms exposed to AgNPs: (**A**) *C. albicans*, (**B**) *P. fermentans*, (**C**) *P. kudriavzevii*, (**D**) *P. bovis* isolate 1, (**E**) *P. bovis* isolate 2, (**F**) *P. bovis* isolate 3, (**G**) *W. pararugosa*, and (**H**) *W. anomalus*. The values shown are average values with standard deviation. C is the control (with no added AgNPs). Statistically significant differences in relation to the control are marked with an asterisk.

The results of the vitality analysis reveal the impact of AgNPs on the metabolic activity and survival rate of the selected yeast and algal strains. Using the PrestoBlue HS assay, changes in cell viability were quantified after treatment with different AgNPs concentrations (Figure 2). The fluorescence data, obtained spectrophotometrically, provided insights into the extent to which various concentrations of AgNPs affected each microorganism’s viability. The following charts depict the measured viability of each microorganism compared to the control, highlighting changes in survival rates at different AgNPs concentrations. The values represent the mean with standard deviation, and statistically significant differences (*p* ≤ 0.001) are marked with an asterisk.

### 2.6. Biofilm Formation

**Figure 3:**
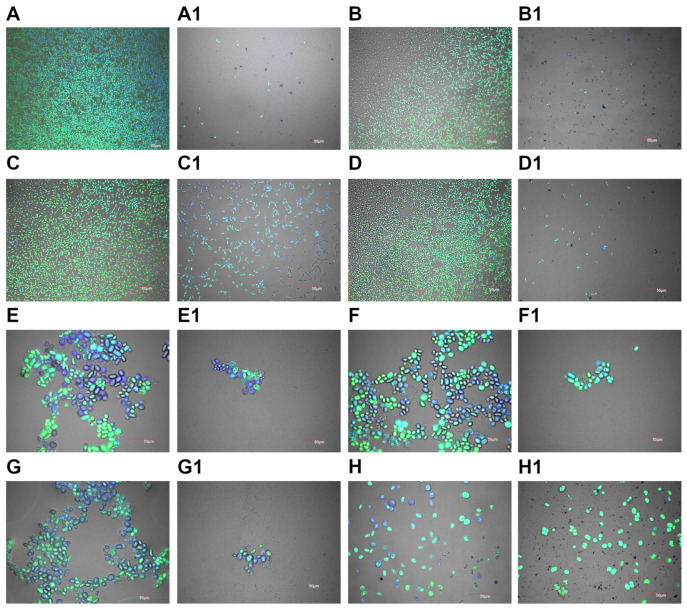
Biofilm formation of (**A**) *C. albicans*, (**B**) *P. fermentans*, (**C**) *P. kudriavzevii*, (**D**) *W. anomalus*, (**E**) *W. pararugosa*, (**F**) *P. bovis* isolate 1, (**G**) *P. bovis* isolate 2, and (**H**) *P. bovis* isolate 3 treated with 10 mg/L of AgNPs (**A1**,**B1**,**C1**,**D1**,**E1**,**F1**,**G1**,**H1**, respectively) using confocal laser microscopy. The blue color is owed to the dye DAPI, green Syto9.

The biofilm formation analysis evaluated the impact of silver nanoparticles (AgNPs) on the growth and biofilm development of various yeast and algal strains. The results indicate that exposure to AgNPs significantly inhibited biofilm formation across most tested strains, with varying degrees of effectiveness observed (Figure 3). In *Candida albicans*, AgNPs treatment drastically inhibited biofilm growth, leaving only a few individual cells visible in the treated sample compared to the control. A similar outcome was observed in *Pichia fermentans*, where biofilm formation was strongly inhibited, resulting in only isolated cells in the treated sample. For *Pichia kudriavzevii*, biofilm growth was significantly inhibited, although slightly more cells were present than other yeast strains. *Wickerhamomyces anomalus* exhibited a marked reduction in biofilm formation, with only a few isolated cells observed in the treated sample. Similarly, *Wickerhamiella pararugosa* displayed a moderate reduction in biofilm formation, as indicated by smaller cell groups in the treated sample compared to the control. In the case of *Prototheca bovis*, the effects of AgNPs treatment varied across the three isolates. Cell clusters for *P. bovis* (Isolate 1) were significantly smaller than in the untreated control, indicating a reduced biofilm-forming capability. A similar reduction in biofilm structure was observed in *P. bovis* (Isolate 2). In contrast, *P. bovis* (Isolate 3) exhibited more cells than the other isolates, suggesting a slightly higher resistance to AgNPs treatment, although biofilm growth was still impaired compared to the control.

### 2.7. Invasion Test

**Figure 4:**
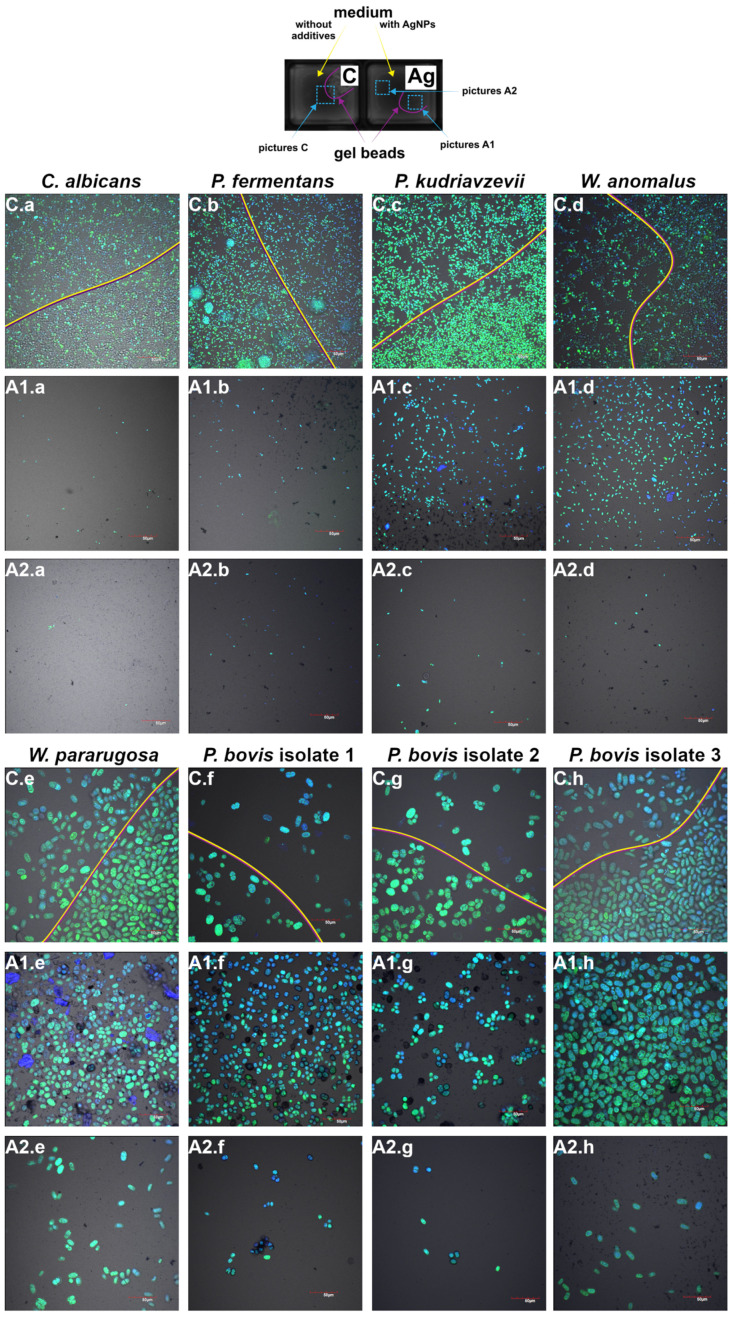
Diagram of the analysis carried out with the marked areas of the pictures (upper part) and exemplary pictures of *C. albicans* (**C.a**, **A1.a**, **A2.a**), *P. fermentans* (**C.b**, **A1.b**, **A2.b**), *P. kudriavzevii* (**C.c**, **A1.c**, **A2.c**), *W. anomalus* (**C.d**, **A1.d**, **A2.d**), *W. pararugosa* (**C.e**, **A1.e**, **A2.e**), *P. bovis* isolate 1 (**C.f**, **A1.f**, **A2.f**), *P. bovis* isolate 2 (**C.g**, **A1.g**, **A2.g**), and *P. bovis* isolate 3 (**C.h**, **A1.h**, **A2.h**) in gel beads. C—control (yellow outside, violet inside gel beads), A—treated with AgNPs, where A1 is part inside gel bead, A2—outside gel bead. The blue color is owed to the dye DAPI, green Syto9.

The invasion test assessed the ability of yeast and algal strains to grow within a semi-solid medium (gel) and migrate beyond its boundary. This test provided insights into the invasive potential of the tested microorganisms and the impact of AgNPs on this process. Figure 4 presents the results. In the control samples, significant invasive growth was observed for most strains, with cells migrating beyond the gel boundary and forming colonies outside. *C. albicans* displayed robust growth in the control, with many colonies forming outside the gel. In contrast, the AgNPs-treated sample exhibited drastically inhibited growth, with only a few isolated cells visible both inside and outside the gel. For *P. fermentans*, substantial invasive growth was observed in the control sample, but the AgNPs-treated sample showed strong inhibition, with only a few cells present. Similarly, *P. kudriavzevii* showed extensive growth and migration in the control, while AgNPs treatment resulted in fewer cells escaping the gel and reduced growth inside the gel. *W. anomalus* exhibited substantial growth and migration in the control, but the AgNPs-treated sample showed reduced cell numbers both within and outside the gel. For *W. pararugosa*, the control sample showed notable growth beyond the gel, whereas AgNPs treatment resulted in fewer migrating cells, though growth within the gel was not significantly affected. Among the *P. bovis* isolates, the control samples displayed varying degrees of invasive growth. Isolate 1 showed modest growth and colony formation outside the gel, but the AgNPs-treated sample had fewer cells and morphological changes, with cells appearing smaller. Isolate 2 demonstrated extensive growth and migration in the control, while the AgNPs-treated sample exhibited reduced cell numbers and morphological alterations. Isolate 3 showed robust growth in the control, with reduced growth and migration in the AgNPs-treated sample but no visible morphological changes. The results show that for the microbial cells, the interior of the gel beads was a more favorable environment than the surrounding medium with AgNPs, which resulted in their low migration and a significantly lower number of cells.

## 3. Discussion

Mastitis caused by yeast and algae is a specific and difficult-to-control type of inflammation of the mammary gland in dairy cattle. Yeast infections are usually associated with poor milking hygiene, contamination of milking equipment, or the use of broad-spectrum antibiotics which disrupt the udder microbiota. Yeasts can enter the udder through the teat canal, where they develop in favorable conditions—such as wet litter or the presence of organic substances—causing chronic inflammation. This type of infection is characterized by the low effectiveness of antibiotic treatment because yeasts are not sensitive to standard antibacterial therapies [22]. Like yeasts, *Prototheca* spp. are also increasingly identified as pathogens causing mastitis. These single-cell organisms are common in moist environments, including water, soil, and manure. *Prototheca* spp. infections are considered endemic on farms where hygiene is difficult to maintain [23,24]. Algal mastitis is often chronic, characterized by the occurrence of multiple inflammatory foci in the mammary gland, leading to permanent damage and a reduced milk quality. Mastitis diagnosed in cow herds causes huge economic losses due to a significantly reduced milk production. The average decrease in milk productivity during mastitis is about 6–7 kg, from a normal productivity of 9 to 10 kg [25]. These infections are difficult to control because *Prototheca* spp. are highly resistant to antibiotics and disinfectants. Such infections often result in the need to remove infected animals from production [19]. This necessitates the use of antimicrobial compounds other than antibiotics. With the help of nanotechnological developments, nanoparticles that can be designed with desired properties have started to appear as promising tools. Nanoparticles have several advantages over traditional antibiotics in combating bacterial infections. Their small size allows them to penetrate biological barriers and directly reach the sites of infection, increasing therapy effectiveness [26]. In addition, their mechanisms of action are multifaceted, including damaging bacterial cell membranes, generating reactive oxygen species, and interfering with pathogen metabolic processes [27]. This diverse approach makes it difficult to develop resistance.

In our research, green synthesis of AgNPs using coffee extract was carried out. In other studies, silver nanoparticles with antimicrobial properties have also been successfully synthesized using differently prepared coffee extracts [18,19,20,21]. There are reports of coffee’s antibacterial properties against, for example, drug-resistant *Vibrio cholerae* [28], *S. typhimurium*, and *E. coli* [29], so trace amounts of coffee extract may have contributed to the antibacterial properties of the synthesized nanoparticles. There are many possible methods to synthesize silver nanoparticles using plant extracts [30]. For example, extracts from plants such as *Ocimum sanctum* (holy basil), *Azadirachta indica* (neem), and *Aloe vera* have been widely used for the green synthesis of silver nanoparticles due to their rich content of bioactive compounds like flavonoids, terpenoids, and phenolic compounds, which act as reducing and stabilizing agents [31,32,33]. The use of plant extracts in the synthesis of silver nanoparticles is advantageous because they are safe for environment, a cheaper option, and do not require the use of toxic chemicals. Additionally, plant extracts contain a variety of phytochemicals that can reduce silver ions (Ag^+^) to silver nanoparticles (Ag^0^) and stabilize them, preventing agglomeration [34].

In the case of coffee extracts, the primary reducing agents are phenolic compounds, such as chlorogenic acids, caffeic acid, and ferulic acid, which are abundant in coffee beans. These compounds have strong antioxidant properties, enabling them to donate electrons and reduce silver ions to metallic silver [35]. Additionally, flavonoids and alkaloids present in coffee extracts may also contribute to the reduction process and provide antimicrobial properties to the synthesized nanoparticles. For instance, flavonoids like quercetin and kaempferol have been shown to enhance the reduction of silver ions and stabilize the resulting nanoparticles [36]. Amino acids, such as cysteine and methionine, can also play a role in the reduction and stabilization of silver nanoparticles due to their thiol and amine functional groups [37]. Therefore, the choice of plant extract and its phytochemical composition significantly influences the synthesis process and the resulting nanoparticles’ properties, including their shape, size, and antimicrobial efficacy. According to the results presented, AgNPs exhibited a spherical shape with a mean hydrodynamic diameter of 86.84 ± 1.99 nm. These results are consistent with those of other researchers [38], in which AgNPs synthesized using spent coffee showed a similar shape regardless of the concentration of the precursor used, which was AgNO_3_. However, the concentration of the precursor may affect the hydrodynamic diameter, and its higher content and spontaneous nucleation contributed to an increase in the growth rate of AgNPs [38]. Nanoparticle size is a crucial parameter determining their toxicity [39]. Smaller nanoparticles, typically in the range of 1–20 nm, exhibit higher toxicity compared to larger particles due to their increased surface area-to-volume ratio, which enhances their reactivity and ability to penetrate biological membranes [40]. For example, studies have shown that silver nanoparticles smaller than 10 nm can easily enter cells and disrupt cellular functions, leading to oxidative stress, DNA damage, and apoptosis [41]. In contrast, larger nanoparticles (above 50 nm) are less toxic because they are less likely to penetrate cell membranes and cause direct damage to intracellular components [42]. Additionally, the size of nanoparticles influences their biodistribution and accumulation in organs, with smaller particles being more likely to accumulate in sensitive tissues such as the liver, spleen, and kidneys, further exacerbating their toxic effects [43]. Therefore, controlling the size of nanoparticles is essential for minimizing their toxicity while maintaining their desired functional properties. The size of the nanoparticles, which approached the 100 nm value, is also related to the zeta potential, which in our research was −21.38 ± 0.94 mV, as the zeta potential is considered to be a parameter that determines the colloidal stability of nanoparticles when it shows values ±30 mV, resulting then in less agglomeration [44]. Similar results were obtained by Liaqat et al. 2022, in which the zeta potential of silver nanoparticles synthesized by green synthesis was found to be −26 mV ± 4.61 mV and −20 mV ± 5.09 mV [26]. High values of zeta potential indicate good colloidal stability, which prevents the formation of aggregates and thus provides greater toxicity [45]. The observed peak between 400 and 500 nm during UV-Vis analysis is typical for AgNPs [19]. Such a peak may result from the absorption of surface plasmon resonances of nanoparticles, which is ascribed to accumulated oscillations of free electrons found on the surface of metallic nanoparticles [46]. However, further examination of AgNPs showed residual carbon, silica, and chloride values, which are presented in LIBS and EDS analysis. The carbon content came from the coffee extract, which is further confirmed by FTIR analysis, which showed the presence of bonds typical of carboxylic acids, ketones, or alkenes. The presence of chlorine is due to the content of chlorogenic acids, which are widely distributed in various plant sources, including coffee beans [38]. The presence of Si in the sample is due to the preparation of the samples for analysis, which were applied to a silicon wafer. [28,29].

Diagnostics of mastitis milk from cows treated with antibiotics revealed the presence of the following microorganisms: *Candida albicans*, *Pichia fermentans*, *Pichia kudriavzevii*, *Wickerhamomyces anomalus*, *Wickerhamiella pararugosa*, and *Prototheca bovis* (3 isolates). *Candida* yeasts may be the main etiological agent of fungal mastitis in dairy cattle. Studies conducted in Mexico identified 20 different *Candida* species in milk samples, the most common of which were *Candida glabrata* and *Candida krusei* [47]. Also frequently found in milk are yeasts of the genus *Pichia*, e.g., *P. kudriavzevii* [48]. However, species such as *W. anomalus* and *W. pararugosa* are rarely diagnosed. *W. anomalus*, not a very common fungus, once included on the QPS (Qualified Presumption of Safety) list of microorganisms by the European Food Safety Authority (EFSA) [49], is now known to cause pediatric infections and infections in immunocompromised individuals [50]. There are now a growing number of reports of *W. anomalus* infections in humans, suggesting that this microorganism has become a new pathogen, although fungemia caused by this species is a rare infection with significant mortality [51]. Moreover, this species has begun to appear among fungal isolates accompanying intramammary infections in dairy cows [52]. Like *W. anomalus*, *Wickerhamiella pararugosa* is an emerging and rare pathogen. It can occur in a variety of organs and biological fluids and has high morbidity and mortality in immunocompromised patients [53]. The pathogen (to an insignificant extent) is sometimes found in dairy products and milk [54]. The algae of the genus *Prototheca* deserve special attention. Within the genus *Prototheca*, two main lineages have been observed, with a dominance of species typically associated with dairy cattle (i.e., *P. ciferrii*, formerly *P. zopfii* gen. 1, *P. blaschkeae*, and one newly derived species, namely *P. bovis*, formerly *P. zopfii* gen. 2) and those associated with humans (i.e., *P. wickerhamii*, *P. cutis*, and *P. miyajii*) [55]. In recent years, cases of mastitis in cows caused by *P. bovis* have been reported all over the world, including Germany, Italy, Poland, Japan, South Korea, China, Brazil, and many other countries and regions [56], indicating a growing problem in controlling this pathogen.

Each of the eight tested microorganisms showed sensitivity to AgNPs synthesized using coffee bean extract. The MIC for *W. pararugosa* and one of *Prototheca bovis* isolate was 10 mg/L, and for *C. albicans*, *P. fermentans*, *P. kudriavzevii*, *W. anomalus* and 2 isolates of *Prototheca bovis* was 20 mg/L. The lethal concentration was 10 mg/L for *P. bovis* (isolate 2), 20 mg/L for *P. fermentans*, *P. kudriavzevii*, *W. pararugosa*, and *P. bovis* (isolate 1), and 40 mg/L for *C. albicans* and *P. bovis* (isolate 3). AgNPs exhibit antifungal activity through mechanisms involving the release of ions, oxidative and nitrosative stress, membrane and cell wall damage, enzymatic activity inhibition, gene expression regulation, decreasing ATP levels, and DNA, protein, and mitochondrial dysfunction [57,58]. With respect to *Prototheca* algae, although research is limited, it has been suggested that silver nanoparticles may disrupt cell membrane functions and metabolic processes, leading to their elimination. Jagielski et al. showed that in all tested *Prototheca* species excluding *P. stagnora*, the MICs (MACs) of AgNPs were within the range of 1–4 mg/L, which translates into a rather high activity of nanosilver, especially when confronted with the activities of clinically available antifungal drugs, some of which used in the treatment of protothecal infections [56]. The activity of AgNPs was also evaluated in the study by Ely et al., obtaining an MIC50 of 30 mg/L and an MIC90 of 60 mg/L [57]. In both of the above cases, changes in the morphology of *Prototheca* cell surface were also noted, such as shrinkage of the protoplast causing the invaginations and detachment of the plasma membrane from the cell wall. The viability of the tested microorganisms determined using the PrestoBlue HS assay indicated a dose-dependent effect of AgNPs on yeast and algae cells. With increasing concentration, the viability of microorganisms decreased. At concentrations of 20 mg/L and higher, low levels of viability or no viability were observed.

Biofilm formation on both biotic and abiotic surfaces is an important virulence factor for pathogenic yeast or filamentous fungi. Biofilms are considered to be up to 1000-fold more resistant to antimicrobials than planktonic forms [59]. It is currently known that not only bacteria and fungi are able to create a more resistant biofilm, but also *Prototheca* spp. [60]. In our research, based on biofilm formation analysis, a significant structure change is visible in most of the tested microorganisms after exposure to AgNPs. In *C. albicans*, *P. fermentans*, and *W. anomalus*, the biofilm structure was almost completely inhibited, and only single cells were visible after treatment with silver nanoparticles. In the 2024 studies of Bahey et al., *C. albicans* was shown to be more susceptible to AgNPs forming a biofilm than other *Candida* species such as *C. tropicalis* or *C. parapisillosis* [61]. Similarly, in a study by Wunnoo et al. in 2021, synthesized nanoparticles using *Eucalyptus citriodora* inhibited the *C. albicans* biofilm by causing surface morphological changes leading to a disruption of the cell wall and membrane [59]. The use of AgNPs in antibiofilm therapy has satisfactory results due to the release of ions that can penetrate the extracellular matrix and react with the cells of microorganisms. Nevertheless, AgNPs with a size greater than 50 nm are believed to have difficulty penetrating the entire biofilm structure [60]. In our research, the AgNPs had a diameter of 86.84 ± 1.99 nm, although those results are due to the agglomerates formed, as indicated by the zeta potential value (−21.38 ± 0.94 mV), which indicates moderately stable nanoparticles, as well as from transmission electron microscopy images which show individual nanoparticles with smaller sizes. What is more, metal nanoparticles are known for ion-releasing [62], which also contributes to a decrease in cell viability.

Bioprinted 3D models of microorganisms and the biofilms they form are more resistant to exposure to antimicrobials than flat 2D cultures. These are models that are more similar to in vivo conditions, and therefore analysis using them allows for the development of more reliable agents to combat pathogenic microorganisms [63]. In our research control groups, the cells spread freely across the medium, moving out of the printed gel fragment. However, there were significantly fewer cells of all microorganisms in the medium with AgNPs, which indicates adverse environmental conditions caused by the presence of AgNPs. Similar results were observed by Wierzbicki et al. (2024), in which silver nanoparticles (in addition to other nanoparticles) also inhibited microbial invasion [64]. These results are consistent with previous ones, in which AgNPs showed high toxicity by limiting the viability of microorganisms and inhibiting biofilm formation, and to conclude—inhibiting the invasion of microorganisms. However, in the studies presented here, a higher dose of AgNPs was used than that allowed by the WHO as a safe intake dose.

The WHO states that 0.1 mg/L of silver in drinking water does not pose a risk to human health and that the level of silver consumed orally by humans over a lifetime can be as high as 10g without observing a harmful effect on health [65]. In the studies presented here, the dose used to inhibit microorganisms was higher than the aforementioned one, which makes the use of silver nanoparticles for action against mastitis in cattle to be considered as a local application. However, optimization of the synthesis process can result in the production of smaller nanoparticle sizes, and their mechanism of action will depend on the exposure dose [39].

## 4. Materials and Methods

### 4.1. Microorganism Strains

The microorganisms used for the study were yeast and algae isolated from mast milk from cows treated with antibiotics from the Mazovian Voivodeship in the spring. The cows were diagnosed with clinical mastitis with a high somatic cell count (SCC) and changes in milk consistency (SCC > 700,000 cells/mL, strong gelation). Each strain was isolated from a separate sample of quart milk and was the only pathogen diagnosed. The milk samples were plated on Sabouraud Agar (Biomaxima, Lublin, Poland) and incubated for 48 h at 37 °C in a microbiological incubator. Individual colonies were then transferred onto new Petri dishes with Sabouraud Agar medium to obtain pure cultures. The identification of yeasts was performed using a MALDI-TOF mass spectrometer (MS) (Bruker, Poznań, Poland), which enables the identification of microorganisms and is based on the *m*/*z* ratio of ions in the tested sample to the ratio in the NCBI (National Center for Biotechnology Information) database. Each NCBI strain has a unique number connected to a specific strain.

To identify *Prototheca* species, partial sequencing of *cytB* gene was performed. A similar method of species identification was carried out by Huilca-Ibarra et al. 2022 [6]. Briefly, genomic DNA was extracted using the GeneMATRIX Environmental DNA & RNA Purification Kit (EURx, Poland). All stages (including additional treatment with lyticase (100 g/mL) (Merck, USA)) were carried out according to the manufacturer’s instructions. The obtained DNA was quantified using a NanoDrop ND-1000 Spectrophotometer (Thermo Fisher Scientific, USA) and stored at 4 °C. For PCR amplification and sequencing, reaction mixtures (30 μL) were prepared, composed of of 1 μL (ca. 10 ng) of template DNA, 18 μL of Color Taq PCR MasterMix (EURx, Poland), 1 μL of each primer cytb-F1(5′-GyGTwGAACAyATTATGAGAG-3′), and *cytb*-R1 (0.2 μM each) (5′-wACCCATAArAArTACCATTCWGG-3′). The PCR cycle included the following thermocycler parameters, 3 min. at 95 °C, followed by 35 cycles of 30 s at 95 °C, 30 s at 50 °C, and 30 s at 72 °C, with a final extension of 5 min. at 72 °C. The amplified products were visualized using agarose gel (1%) electrophoresis, stained with ethidium bromide (5 mg/L), and exposed to UV light. The amplicons were purified using the Short DNA Clean-Up (EURx, Poland) and directly sequenced with the same primers as those used for the amplification. The resulting sequences were assembled in the Clone Professional Manager Suite 8 program and subsequently compared with the reference sequences of the Prototheca-ID web application and the NCBI GenBank database (Supplementary Material).

Species that were repeaters or contaminations after identification were discarded for further study. According to the preference of the study material, 8 species were selected after identification of all isolates (including 3 *Prototheca* spp.).

### 4.2. Synthesis and Physicochemical Characterization of Silver Nanoparticles

Green synthesis of silver nanoparticles was performed using AgNO_3_ as a precursor and coffee extract as a source of reducing agents and stabilizers. A total of 10 g of the dried, roasted seeds were finely crushed and stirred with 90 mL of deionized water at 90 °C for 1 h and filtered by a Whatman No. 1 filter paper to obtain the extract. The filtrate was further used as a reducing agent and also as a stabilizer. A total of 0.05 g AgNO_3_ was dissolved in 45 mL of deionized water, and then 5 mL of coffee bean extract was added. The reaction mixture was stirred using a magnetic stirrer (100 rpm/min) at 25 °C for 2 h. Color changes were observed from light brown to dark brown, indicating that the reaction has terminated. The reduced mixture was purified by centrifugation thrice at 4000 rpm for 10 min. The supernatant was transferred to new centrifuge tubes to sediment the remaining nanoparticles. The obtained sample was dried in a laboratory oven at 60 °C. Nanoparticle hydrocolloids were prepared by suspending dried AgNPs (0.05 g) in ultrapure water (50 mL) to form a stock solution with a concentration of 1000 mg/L.

### 4.3. Physicochemical Analysis of Nanoparticles

AgNPs were placed onto Cu grids (3 mm 200 mesh Cu grids, Formvar, Agar Scientific, Stansted, UK) and observed using a transmission electron microscope (TEM, JEM-1220, JEOL, Tokyo, Japan) operated at a voltage of 80 keV. Zeta potential and size distribution of AgNPs were measured using a Zetasizer ZSP (Malvern Instruments Ltd., Malvern, UK) at 25 °C using the laser dynamic scattering electrophoretic method. Before measurements, AgNPs were sonicated at 500 W and 20 kHz for 2 min using a VC 505 Ultrasonic Liquid Processor with a cup horn (Sonics & Materials, Newtown, CT, USA). All measurements were performed in triplicate.

For structural studies EDX, LIBS, and FTIR-ATR, 0.05 mL of AgNPs was applied at the same location seven times using an automatic laboratory pipette on a silicon wafer (Prime CZ-Si wafer 24.5 ± 0.3 mm diameter, thickness = 200 ± 25 µm, (100), 1-side polished CZ-Si wafer WSA10200250B1314XNN1), and then placed in a vacuum dryer (Vacucell 22L) at 40 °C for about 0.5 h.

- LIBS: Studies using laser induced breakdown spectroscopy (LIBS) enabled the identification of elements present in dried suspensions of AgNPs. A laser beam focused on the surface of the dried suspension caused its ablation, followed by heating and ionizing the resulting vapors and generation of plasma. The plasma thus generated was a source of strong radiation, specific to the atoms in the suspension. The study was conducted in the experimental setup shown in the Nasiłowska et al. 2023 [15]. The plasma was generated using a Quantel pulsed Nd/YAG laser, model Brio. The experiments were carried out with radiation at a wavelength of 1064 nm according to the parameters listed in Table 5.
- EDS: The elemental composition of the dried suspensions of AgNPs was also determined using an EDX detector (energy dispersive X-ray spectrometer, FEI, (FEI, Hillsboro, OR, USA) coupled to a scanning electron microscopy (SEM) (Quanta 250 FEG SEM, FEI, Hillsboro, OR, USA). The accelerating voltage was 30 kV, while the spot was 6.
- FTIR-ATR: The functional groups of the dried suspensions were determined by Fourier-transform infrared spectroscopy (FTIR). The dried suspensions of AgNPs were analyzed by FTIR (Nicolet IS50, FTIR, ThermoFisher Scientific, Waltham, MA, USA). Samples were measured three times using ATR (total internal reflection) in the 400–4000 cm^−1^ range with a resolution of 4 cm^−1^ and for 64 scans.

### 4.4. Minimal Inhibitory Concentration (MIC)

The glucose chloramphenicol broth (Biomaxima, Lublin, Poland) dilution was used to study the antimicrobial efficacy of AgNPs by evaluating the visible growth of microorganisms in the broth. Serial dilutions of AgNPs in concentrations of 320, 160, 80, 40, 20, 10, 5, 2.5, 1.25, and 0.625 mg/L with adjusted microorganisms’ concentration 0.5 OD (1.0–5.0 × 10^6^ cfu/mL, OD_570nm_) were used to determine the MIC in glucose chloramphenicol broth. The control contained only inoculated broth and was incubated for 24 h at 37 °C. The MIC endpoint is the lowest concentration of silver nanoparticles with no visible growth.

### 4.5. Minimal F/A Concentration (MFC/MAC)

After the MIC determination of the AgNPs, solutions from all the wells were seeded on dichloran, Bengal red, chloramphenicol (DRBC, Biomaxima, Lublin, Poland) agar plates and incubated for 24 h at 37 °C. When no growth of the microorganism population is observed at the lowest concentration of an antimicrobial agent, it is termed as the MFC and MAC endpoint. This was performed by observing pre- and post-incubated DRBC agar plates for the presence or absence of microorganisms.

### 4.6. Viability Analysis

Metabolic activity was measured using the PrestoBlue HS assay (ThermoFischer, Waltham, MA, USA) to assess cell viability. PrestoBlue is a reagent based on resazurin, which is reduced to resorufin, a red fluorescent compound that can be measured quantitatively to determine viability [66].

Microorganism suspensions were placed in a 96-well plate in the culture medium (glucose chloramphenicol broth) containing silver nanoparticles at concentrations of 320, 160, 80, 40, 20, 10, 5, 2.5, 1.25, and 0.625 mg/L and incubated for 24 h. The positive control was a microorganism suspension without adding AgNPs, while negative control groups consisted of medium without microorganisms. After 24 h of incubation, 10 μL of PrestoBlue reagent was added directly to the existing culture medium in each well. After 15 min of incubation, the fluorescence was measured using spectrophotometry (excitation wavelength 560 nm, emission wavelength 590 nm). Results were calculated as a percentage of viability in relation to the control samples reduced by negative control values.

### 4.7. Biofilm Formation

To evaluate the effect of AgNPs on biofilm formation, microorganisms were cultured in a µ-Slide 8-well plate, which provided a controlled environment for biofilm development. Each well was inoculated with a standardized concentration of microorganisms in a culture medium, with AgNPs added at concentrations of 10 mg/L. The control wells contained microorganisms in a medium without AgNPs. After a 48 h incubation period at 37 °C, wells were gently washed with phosphate-buffered saline (PBS) to remove planktonic cells, leaving only those attached within the biofilm. Cells were then fixed with 4% paraformaldehyde for 15 min to stabilize its structure. Following fixation, the formalin was carefully removed, and the biofilm biomass was stained with 4′,6-diamidino-2-phenylindole (DAPI) and SYTO9 solution for 15 min and then poured with 80% glycerol. Samples were analyzed with the use of confocal microscopy (FV-1000; Olympus Corporation, Tokyo, Japan) with a laser wavelength of 405 nm for DAPI and 488 nm for Syto9.

### 4.8. Invasion Test

Determination of the microorganism’s ability to invade from alginate beads to a medium containing AgNPs was assessed by confocal microscopy [64].

Microorganism suspensions were first prepared to an optical density of 0.5. Then, 50 μL of each suspension was mixed with alginate. Droplets of 10 μL of the alginate–microorganism mixture were bioprinted onto a plate (µ-Slide 8-well plates) and crosslinked with magnesium chloride (MgCl₂) to form alginate gel beads. Following crosslinking, microbiological media were added to the wells, with one set containing only medium as a control and the other containing medium with the addition of AgNPs (final concentration 10 mg/L). After 24 h of incubation (37 °C), plates were washed twice with PBS to remove non-adherent microorganisms, and the remaining ones were stained with DAPI and Syto9 fluorescent dyes for 30 min.

Samples were analyzed using confocal microscopy (FV-1000; Olympus Corporation, Tokyo, Japan) with a laser wavelength of 405 nm for DAPI and 488 nm for Syto9.

### 4.9. Statistical Analysis

The results of the toxicity tests of silver nanoparticles (AgNPs) on selected non-bacterial pathogens were analyzed using one-way analysis of variance (ANOVA) in GraphPad Prism 5 software (version 5.00, San Diego, CA, USA). Tukey’s post hoc test was used for group comparisons. Results were expressed as mean values with standard deviations, and differences with a *p*-value ≤ 0.05 were considered statistically significant.

## 5. Conclusions

Green synthesis of AgNPs using coffee bean extract allows for obtaining nanoparticles with satisfactory antimicrobial properties. The obtained nanoparticles inhibit the viability of yeast and also algae *Prototheca* spp., prevent biofilm formation, and inhibit the invasion of microorganisms. The results represent an innovative, satisfactory solution in the context of potential treatment of mastitis in cattle caused by microorganisms other than bacteria, of which there are far fewer ways to combat.

## Figures and Tables

**Table 1:** Microorganisms identified using the MALDI-TOF MS, including score value and NCBI identifier.

Matched Pattern	Score Value	NCBI Identifier
*Candida albicans*	2.05	5476
*Pichia fermentans*	2.21	53655
*Pichia kudriavzevii*	2.06	4909
*Wickerhamomyces anomalus*	2.08	4927
*Wickerhamiella pararugosa*	2.18	49331

**Table 2:** Microorganisms identified using the partial sequencing, including GenBank identifier.

Strain	Species	Material	Preliminary Indetification	GenBank
PRO3	*Prototheca bovis* (Isolate 1)	Quarter milk	Phenotypic Characteristics	PQ151373
PRO7	*Prototheca bovis* (Isolate 2)	Quarter milk	Phenotypic Characteristics	PQ1513734
PRO32	*Prototheca bovis* (Isolate 3)	Quarter milk	Phenotypic Characteristics	PQ151375

**Table 3:** MIC values of synthesized AgNPs for isolated microorganisms.

Concentration (mg/L)	*C. albicans*	*P. fermentans*	*P. kudriavzevii*	*W. anomalus*	*W. pararugosa*	*P. bovis* Isolate 1	*P. bovis* Isolate 2	*P. bovis* Isolate 3
320	−	−	−	−	−	−	−	−
160	−	−	−	−	−	−	−	−
80	−	−	−	−	−	−	−	−
40	−	−	−	−	−	−	−	−
20	−	−	−	−	−	−	−	−
10	+	+	+	+	+	+	+	+
5	+	+	+	+	+	+	+	+
2.5	+	+	+	+	+	+	+	+
1.25	+	+	+	+	+	+	+	+
0.625	+	+	+	+	+	+	+	+
PC	+	+	+	+	+	+	+	+

Note: “+” indicates no growth inhibition, and “−” indicates inhibition of growth. PC is positive control.

**Table 4:** MFC/MAC values of synthesized AgNPs for isolated microorganisms.

Concen-tration (mg/L)	*C. albi-cans*	*P. fer-mentans*	*P. kudriavzevii*	*W. anomalus*	*W. pararugosa*	*P. bovis* Isolate 1	*P. bovis* Isolate 2	*P. bovis* Isolate 3
320	−	−	−	−	−	−	−	−
160	−	−	−	−	−	−	−	−
80	−	−	−	−	−	−	−	−
40	−	−	−	−	−	−	−	−
20	+	−	−	+	−	−	−	+
10	+	+	+	+	+	+	+	+
5	+	+	+	+	+	+	+	+
2.5	+	+	+	+	+	+	+	+
1.25	+	+	+	+	+	+	+	+
0.625	+	+	+	+	+	+	+	+
PC	+	+	+	+	+	+	+	+

Note: “+” indicates no growth inhibition, and “−” indicates inhibition of growth. PC is positive control.

**Table 5:** Parameters used during the LIBS study.

Parameter	Value
Accumulation (shots number)	1
Gate width, ns	500
Gate delay, ns	500
Pulse energy, mJ	46

## Data Availability

The original contributions presented in this study are included in the article/Supplementary Materials. Further inquiries can be directed to the corresponding author(s).

## Supplementary Materials

The following supporting information can be downloaded at: https://www.mdpi.com/article/10.3390/molecules30061369/s1. File S1: Prototheca bovis isolate PRO 3 cytochrome b (cytb) gene, partial cds; mitochondrial. Prototheca bovis isolate PRO 7 cytochrome b (cytb) gene, partial cds; mitochondrial. Prototheca bovis isolate PRO 32 cytochrome b (cytb) gene, partial cds; mitochondrial.
