# Supplementary material for: Green Nanoparticle Synthesis in the Application of Non-Bacterial Mastitis in Cattle
Source: Molecules. 2025 Mar 18;30(6):1369. doi: 10.3390/molecules30061369 (PMC11944971; doi:10.3390/molecules30061369)
Supplement: Supplementary file 1 [file molecules-30-01369-s001.zip › molecules-3475437-supplementary.pdf]

Supplementary material

Green nanoparticle synthesis in the application of non-bacterial mastitis in cattle

Michał Motrenko [1], Agata Lange [1*], Aleksandra Kalińska [2], Marcin Gołębiewski [2], Małgorzata Kunowska-Slósarz [2], Barbara Nasiłowska [3], Joanna Czwartos [3], Wojciech Skrzeczanowski [3], Aleksandra Orzeszko-Rywka [4], Tomasz Jagielski [5], Anna Hotowy [1], Mateusz Wierzbicki [1], Sławomir Jaworski [1]

[1]Department of Nanobiotechnology, Institute of Biology, Warsaw University of Life Sciences, Warsaw, Poland
[2]Animal Breeding Department, Warsaw University of Life Sciences, Warsaw, Poland
[3] Institute of Optoelectronics, Military University of Technology, Warsaw, Poland
[4] Department of Plant Physiology, Institute of Biology, Warsaw, Poland
[5] Department of Applied Microbiology, Institute of Microbiology, Faculty of Biology, University of Warsaw, Warsaw, Poland
* Correspondence: agata_lange1@sggw.edu.pl; Tel.: +22 5935809

1) *Prototheca bovis* isolate PRO 3 cytochrome b (cytb) gene, partial cds; mitochondrial

GenBank: PQ151373.1

LOCUS PQ151373 599 bp DNA linear PLN 09-SEP-2024

DEFINITION Prototheca bovis isolate PRO 3 cytochrome b (cytb) gene, partial cds; mitochondrial.

ACCESSION PQ151373

SOURCE mitochondrion Prototheca bovis

ORGANISM Prototheca bovis

Eukaryota; Viridiplantae; Chlorophyta; core chlorophytes;

Trebouxiophyceae; Chlorellales; Chlorellaceae; Prototheca

FEATURES Location/Qualifiers

source 1..599

/organism="Prototheca bovis"

/organelle="mitochondrion"

/mol_type="genomic DNA"

/isolate="PRO 3"

/isolation_source="bovine quarter milk"

/host="cow"

/db_xref="taxon:2509265"

/geo_loc_name="Poland"

gene <1..>599

/gene="cytb"

CDS <1..>599

/gene="cytb"

/codon_start=3

/product="cytochrome b"

/protein_id="XFF05946.1"

/translation="VEGGWFLRYMHANGASMFFIVVYSHMFRGLYFSSYSSPRELTWI

AGVAILLLMIITAFIGYVLPWGQMSFWGATVITSLASAIPVVGNSIVTWLWGGFSIDN
ATLNRFFSLHYLLPFVIAGLSIVHIAALHQYGSNNPLGINAKTDKISFYPYFYVKDLF

GWTIFAFFFAYFIYYNPNLLGHPDNYIPANPMSTPAHIV"

ORIGIN

1 atgttgaagg tggctggttt ttacgttaca tgcatgcaaa tggtgcaagt atgtttttta

61 ttgtagtata ttctcatatg tttagaggat tatacttttc tagttattca agtcctcgtg

121 aattaacttg gattgcaggt gtagctattt tattattaat gattattact gcatttatcg

181 gttacgtatt accttggggt caaatgagct tttggggtgc tacagtaatt acaagcttag

241 ctagtgcaat tcctgtggtg ggtaatagta ttgtaacttg gctatggggt ggtttctcta

301 ttgataacgc aacattaaac cgtttcttta gtttacatta tttattacca tttgtaattg

361 ctggattatc tattgttcat attgcagcat tacatcaata tggttcaaat aacccttta g

421 gtattaatgc aaaaactgat aaaattagtt tttatccata tttttatgtg aaagatttat

481 ttggttggac tatttttgct tttttctttg catattttat ctattataat ccaaacttat

541 taggtcaccc tgataattac attcctgcga accctatgtc aactccagca catattgta

//

2) Prototheca bovis isolate PRO 7 cytochrome b (cytb) gene, partial cds; mitochondrial

GenBank: PQ151374.1

LOCUS PQ151374 599 bp DNA linear PLN 09-SEP-2024

DEFINITION Prototheca bovis isolate PRO 7 cytochrome b (cytb) gene, partial cds; mitochondrial.

ACCESSION   PQ151374

SOURCE   mitochondrion Prototheca bovis

ORGANISM Prototheca bovis

Eukaryota; Viridiplantae; Chlorophyta; core chlorophytes;

Trebouxiophyceae; Chlorellales; Chlorellaceae; Prototheca.

FEATURES   Location/Qualifiers

source   1..599

/organism="Prototheca bovis"

/organelle="mitochondrion"

/mol_type="genomic DNA"

/isolate="PRO 7"

/isolation_source="environmental sample from dairy herd"

/db_xref="taxon:2509265"

/geo_loc_name="Poland"

gene   <1..>599

/gene="cytb"

CDS   <1..>599

/gene="cytb"

/codon_start=3

/product="cytochrome b"

/protein_id="XFF05947.1"

/translation="VEGGWFLRYMHANGASMFFIVVYSHMFRGLYFSSYSSPRELTWI
AGVAILLLMIITAFIGYVLPWGQMSFWGATVITSLASAIPVVGNSIVTWLWGGFSIDN
ATLNRFFSLHYLLPFVIAGLSIVHIAALHQYGSNNPLGINAKTDKISFYPYFYVKDLF

GWTIFAFFFAYFIYYNPNLLGHPDNYIPANPMSTPAHIV"

ORIGIN

1 atgttgaagg tggctggttt ttacgttaca tgcatgcaaa tggtgcaagt atgtttttta

61 ttgtagtata ttctcatatg tttagaggat tatacttttc tagttattca agtcctcgtg

121 aattaacttg gattgcaggt gtagctattt tattattaat gattattact gcatttatcg

181 gttacgtatt accttggggt caaatgagct tttggggtgc tacagtaatt acaagcttag

241 ctagtgcaat tcctgtggtg ggtaatagta ttgtaacttg gctatggggt ggtttctcta

301 ttgataacgc aacattaaac cgtttcttta gtttacatta tttattacca tttgtaattg

361 ctggattatc tattgttcat attgcagcat tacatcaata tggttcaaat aaccctttag

421 gtattaatgc aaaaactgat aaaattagtt tttatccata tttttatgtg aaagatttat

481 ttggttggac tatttttgct tttttctttg catattttat ctattataat ccaaacttat

541 taggtcaccc tgataattac attcctgcga accctatgtc aactccagca catattgta

//

3) **Prototheca bovis isolate PRO 32 cytochrome b (cytb) gene, partial cds; mitochondrial**

GenBank: PQ151375.1

LOCUS PQ151375 599 bp DNA linear PLN 09-SEP-2024

DEFINITION Prototheca bovis isolate PRO 32 cytochrome b (cytb) gene, partial cds; mitochondrial.

ACCESSION PQ151375

SOURCE mitochondrion Prototheca bovis

ORGANISM Prototheca bovis

Eukaryota; Viridiplantae; Chlorophyta; core chlorophytes;

Trebouxiophyceae; Chlorellales; Chlorellaceae; Prototheca.

FEATURES Location/Qualifiers

source 1..599

/organism="Prototheca bovis"

/organelle="mitochondrion"

/mol_type="genomic DNA"

/isolate="PRO 32"

/isolation_source="bovine quarter milk"

/host="cow"

/db_xref="taxon:2509265"

/geo_loc_name="Poland"

gene <1..>599

/gene="cytb"

CDS <1..>599

/gene="cytb"

/codon_start=3

/product="cytochrome b"

/protein_id="XFF05948.1"

/translation="VEGGWFLRYMHANGASMFFIVVYSHMFRGLYFSSYSSPRELTWI
AGVAILLLMIITAFIGYVLPWGQMSFWGATVITSLASAIPVVGNSIVTWLWGGFSIDN
ATLNRFFSLHYLLPFVIAGLSIVHIAALHQYGSNNPLGINAKTDKISFYPYFYVKDLF

GWTIFAFFFAYFIYYNPNLLGHPDNYIPANPMSTPAHIV"

ORIGIN

1 atgttgaagg tggctggttt ttacgttaca tgcatgcaaa tggtgcaagt atgtttttta

61 ttgtagtata ttctcatatg tttagaggat tatacttttc tagttattca agtcctcgtg

121 aattaacttg gattgcaggt gtagctattt tattattaat gattattact gcatttatcg

181 gttacgtatt accttggggt caaatgagct tttggggtgc tacagtaatt acaagcttag

241 ctagtgcaat tcctgtggtg ggtaatagta ttgtaacttg gctatggggt ggtttctcta

301 ttgataacgc aacattaaac cgtttcttta gtttacatta tttattacca tttgtaattg

361 ctggattatc tattgttcat attgcagcat tacatcaata tggttcaaat aacccttag

421 gtattaatgc aaaaactgat aaaattagtt tttatccata tttttatgtg aaagatttat

481 ttggttggac tatttttgct tttttctttg catattttat ctattataat ccaaacttat

541 taggtcaccc tgataattac attcctgcga accctatgtc aactccagca catattgta

//
